# Acute clouding of a trifocal intraocular lens with spontaneous resolution: a case report

**DOI:** 10.1186/s12886-019-1216-9

**Published:** 2019-10-17

**Authors:** Tao Zhang, Shaowei Li, Chang Liu, Ruihua Zhao, Chenghe Chang, Na Han

**Affiliations:** 1 Department of Ophthalmology, Aier School of Ophthalmology, Central South University, 932 South Lushan, Yuelu District Changsha, 410083 People’s Republic of China; 2 The First Affiliated Hospital of Shantou University Medical College, Shantou, 515041 Guangdong China; 3 Department of Ophthalmology, Beijing Aier-Intech Eye Hospital, NO.12 Panjiayuan Nanli, Chaoyang District Beijing, 100021 People’s Republic of China

**Keywords:** AT LISA tri 839MP, Clouding, Clearing

## Abstract

**Background:**

Opacification of hydrophobic and hydrophilic intraocular lenses (IOLs) has been reported. Herein, we report a case of spontaneous resolution of opacification following acute clouding of a trifocal IOL, which consisted of hydrophilic acrylic material (25%) with hydrophobic surface properties, occurring in a cold region in the winter season.

**Case presentation:**

A young adult with bilateral radiation cataract underwent phacoemulsification using a femtosecond laser and implantation of a trifocal IOL. The trifocal IOL was delivered to the operating theatre 30 min before the surgery. The outside temperature was approximately − 7 °C. The IOL package was warmed using a radiator at approximately 35 °C for 15 min. After the optical region was implanted in the eye, cloudiness was observed, which persisted throughout the operation. Complete clearing of the IOL was apparent after three postoperative hours.

**Conclusion:**

In this case, rapid opacification and clearing of the IOL suggested an acute and transient process. IOLs should be stored and shipped at a constant temperature, and sudden temperature fluctuations should be avoided, especially in the colder seasons.

## Background

Trifocal intraocular lenses (IOLs) provide visual acuity similar to the normal visual acuity of the human eye. However, IOL opacification is a severe complication of cataract surgery, resulting in blurred vision and explantation of both hydrophilic and hydrophobic IOLs [1–3]. Delayed IOL opacification is known as “pseudocataract” [4]. Intraoperative acute clouding of acrylic hydrophilic IOLs with spontaneous resolution has been reported [5]. Herein, we report a case of early intraoperative opacification of a trifocal IOL (AT LISA tri 839MP, Carl Zeiss Meditec, Jena, Germany) with resolution within three postoperative hours.

## Case presentation

A 25-year-old man presented with bilateral cataract. He underwent cataract surgery in the right eye with a femtosecond laser to correct astigmatism and implantation of a + 15.0-dioptre trifocal IOL (AT LISA tri 839MP, Carl Zeiss). The operation was performed by the author Dr. Li on January 16, 2018.

Drugs were administered perioperatively following the standard protocol. Docking of the patient’s eye was performed; femtosecond laser instruments were used to perform anterior capsulorhexis and sequential nuclear division; a corneoscleral incision was made with a knife to strengthen the coalescence. Subsequently, phacoemulsification was performed uneventfully under a microscope.

**Fig. 1:**
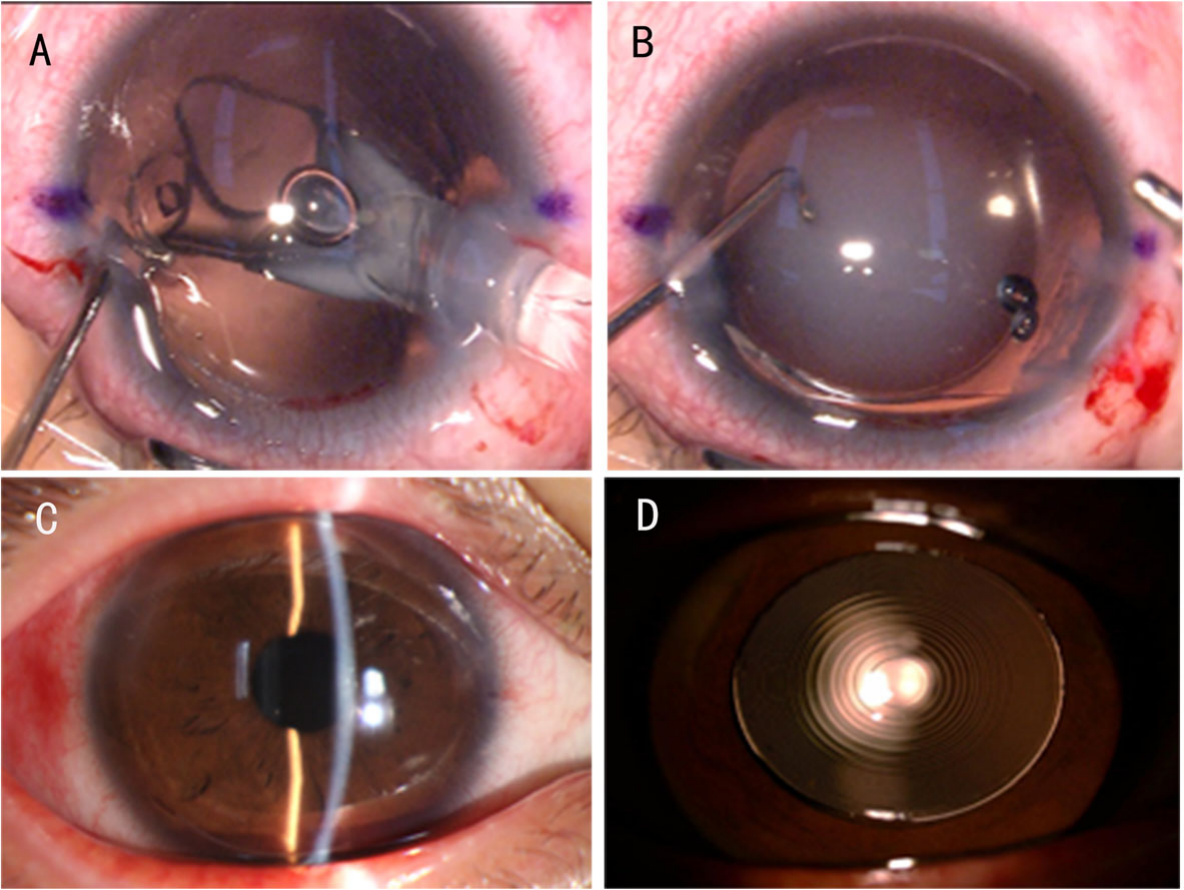
**a** Clouding of the intraocular lens. Cloudiness was observed following implantation of the optical region in the eye. **b** Clouding of the intraocular lens. Cloudiness was observed following complete insertion of the intraocular lens in the anterior chamber of the eye and it persisted throughout the operation. **c** Intraocular lens at three postoperative hours. The intraocular lens was transparent, showing complete resolution of clouding, at three postoperative hours. **d** Intraocular lens on postoperative day 1. No residual opacification or structural changes are observed in the trifocal IOL on postoperative day 1

Because of delay in shipping of the IOL package from another city, the IOL was delivered to the operating theatre only 30 min before the start of the operation. The outside temperature was approximately − 7 °C; therefore, the IOL package was warmed using a radiator at a temperature of approximately 35 °C for 15 min. Once the optical region was implanted in the eye, cloudiness was observed, which persisted throughout the operation. (Fig. 1a, b). Considering the possibility of spontaneous clearing, which was previously reported in an IOL of another brand and following consultation from an IOL specialist, the changes were monitored. Three hours postoperatively, the IOL became completely transparent, and the visual acuity was restored to 0.3 logarithm of the Minimal Angle of Resolution (Fig. 1c. At the scheduled follow-ups on postoperative days 1 and 3, no residual opacification or structural changes in the trifocal IOL (Fig. 1d) were observed, and the patient’s uncorrected visual acuity was 20/20.

## Discussion

We reported a case of spontaneous resolution of trifocal IOL opacification following acute clouding. Although acute clouding of IOLs have been described by Liu et al. [6], both lenses in that study remained cloudy postoperatively for l h and 8 min in vivo, respectively. Therefore, a new IOL was implanted. However, the IOL in our case exhibited clearing within three postoperative hours.

After conducting photochemistry with the 400-420 nm wavelength of ultraviolet rays for 5 months, the patient suffered from bilateral radiation cataract. Toto et al. [7] reported that inflammatory infiltrates could be detected immunohistochemically in the anterior segment of both the eyes with cataract, induced with ultraviolet-B radiation exposure. However, there are no reports describing acute clouding associated with inflammation in bilateral radiation cataracts.

Pseudocataract could occur spontaneously because of calcium and phosphate accumulation, which results in hydroxyapatite crystal formation [8, 9]. Such delayed IOL opacification usually occurs after a few postoperative months [4], unlike our case. Tyagi et al. [1], Dhoot et al. [5], Liu et al. [6], and Helvaci et al. [10] reported that intraoperative clouding of hydrophilic and hydrophobic IOLs could be caused by sudden changes in temperature. In addition, the acute clouding reported by Tyagi et al. [1] lasted for approximately 3 h and disappeared spontaneously, and the opacification reported by Helvaci [10] resolved the following day. The opacification of two trifocal IOLs of the same company as ours reported by Liu et al. [8] persisted for 1 h and 8 min, respectively, without any equilibration in vivo, and the second IOL became transparent 5 min later in vitro. Eventually, both IOLs were explanted, and an IOL of another brand (ZCB200, Allergan, Dublin, Ireland) was implanted. Severe fluctuations in temperature were also observed in the IOL in our case. However, acute clouding disappeared within three postoperative hours. Based on the aforementioned cases, the manufacturer’s instructions state that the IOL should be at room temperature at the time of surgery to avoid temporary clouding of the lens optic following implantation. However, the time of resolution of the temporary clouding has not been elucidated, and there have been no reports of the spontaneous resolution of clouding of hybrid hydrophilic and hydrophobic IOLs. Considering that the package and storage buffer were intact, the acute IOL opacification could have resulted from sudden changes in temperature; therefore, we observed the condition and did not perform explantation. Regarding the formation of acute clouding, we hypothesized that the air inside the IOL was released when the temperature suddenly increased, resulting in microbubbles. Microbubbles and light refraction led to the appearance of clouding. When air dissolution reached an equilibrium both in water and the IOL, the clouding disappeared spontaneously.

The IOL in our case (ATLISA tri 839MP) illustrated the effect of environment on the transparency of hydrophilic acrylic material (25%) with hydrophobic surface properties in cataract surgery. We could not confirm whether or not acute clouding could alter the mechanical, chemical, or geometric characteristics of the IOL polymer. Long-term changes in the IOL should be investigated in the subsequent follow-ups. The appropriate temperature should be maintained during the storage and transport of IOLs to avoid transient clouding due to temperature fluctuations. IOLs should be handled carefully by the surgeon and kept at room temperature for some time before implantation to prevent clouding.

## Conclusion

IOL clouding can resolve spontaneously; therefore, the patient should be observed.

## Data Availability

The datasets from the current study can be obtained on request from the corresponding author.

## Abbreviation

IOL: Intraocular lens
